# The impact of an online patient decision aid for women with breast cancer considering immediate breast reconstruction: study protocol of a multicenter randomized controlled trial

**DOI:** 10.1186/s12911-019-0873-1

**Published:** 2019-08-19

**Authors:** Jacqueline A. ter Stege, Leonie A. E. Woerdeman, Daniela E. E. Hahn, Martine A. van Huizum, Frederieke H. van Duijnhoven, Jacobien M. Kieffer, Valesca P. Retèl, Kerry A. Sherman, Arjen J. Witkamp, Hester S. A. Oldenburg, Eveline M. A. Bleiker

**Affiliations:** 1 Division of Psychosocial Research and Epidemiology, Netherlands Cancer Institute, Plesmanlaan 121, 1066CX Amsterdam, The Netherlands; 2 Department of Plastic and Reconstructive Surgery, Netherlands Cancer Institute, Plesmanlaan 121, 1066CX Amsterdam, The Netherlands; 3 Department of Psychosocial Counseling, Netherlands Cancer Institute, Plesmanlaan 121, 1066CX Amsterdam, The Netherlands; 4 Department of Surgical Oncology, Netherlands Cancer Institute, Plesmanlaan 121, 1066CX Amsterdam, The Netherlands; 5 Centre for Emotional Health, Department of Psychology, Macquarie University, Balaclava Rd, North Ryde, Sydney, NSW 2019 Australia; 6 Department of Surgery, University Medical Center Utrecht, Heidelberglaan 100, 3584 CX Utrecht, The Netherlands; 7 Family Cancer Clinic, Netherlands Cancer Institute, Plesmanlaan 121, 1066CX Amsterdam, The Netherlands; 8 Department of Clinical Genetics, Leiden University Medical Center, Albinusdreef 2, 2333 ZA Leiden, The Netherlands

**Keywords:** Breast cancer, Breast reconstruction, Patient decision aid, Decisional conflict, Randomized controlled trial

## Abstract

**Background:**

Most breast cancer patients undergoing mastectomy are candidates for breast reconstruction. Deciding about breast reconstruction is complex and the preference-sensitive nature of this decision requires an approach of shared decision making between patient and doctor. Women considering breast reconstruction have expressed a need for decision support. We developed an online patient decision aid (pDA) to support decision making in women considering immediate breast reconstruction. The primary aim of this study is to assess the impact of the pDA in reducing decisional conflict, and more generally, on the decision-making process and the decision quality. Additionally, we will investigate the pDA’s impact on health outcomes, explore predictors, and assess its cost-effectiveness.

**Methods:**

A multicenter, two-armed randomized controlled trial (1:1) will be conducted. Women with breast cancer or ductal carcinoma in situ who will undergo a mastectomy and are eligible for immediate breast reconstruction will be invited to participate. The intervention group will receive access to the online pDA, whereas the control group will receive a widely available free information leaflet on breast reconstruction. Participants will complete online questionnaires at: baseline (T0), 1 week after consultation with a plastic surgeon (T1), and 3 (T2) and 12 months (T3) after surgery. The primary outcome is decisional conflict. Secondary outcomes include other measures reflecting the decision-making process and decision quality (e.g., decision regret), patient-reported health outcomes (e.g., satisfaction with the breasts) and costs.

**Discussion:**

This study will provide evidence about the impact of an online pDA for women who will undergo mastectomy and are deciding about breast reconstruction. It will contribute to the knowledge on how to optimally support women in making this difficult decision.

**Trial registration:**

This study is retrospectively registered at ClinicalTrials.gov (NCT03791138).

## Background

Breast cancer is the most prevalent type of cancer amongst women [1, 2]. In Western European countries, approximately one in eight women will develop breast cancer over the course of her lifetime [1, 3]. In The Netherlands alone, more than 14 000 women developed breast cancer in 2018 [1]. Approximately 60–70% of all breast cancer patients undergo breast-conserving surgery (BCS) in which the tumor and a margin of surrounding breast tissue is removed, conserving breast shape as much as possible [4–6]. However, another 30–40% of breast cancer patients undergo a mastectomy, in which all breast tissue is removed and breast contour therefore is lost [4–7]. Breast cancer surgery, especially mastectomy, can negatively impact psychosocial outcomes such as body image, sexual functioning and health-related quality of life [8–11]. To restore breast contour, and potentially reduce the negative psychosocial impact of mastectomy, women may opt for breast reconstructive surgery.

Breast reconstruction can be performed immediate at the time of mastectomy, or delayed, typically when treatment for breast cancer is completed. Furthermore, there are several types of breast reconstruction (implant-based, autologous, and a combination of both). All options have their pros and cons, and personal values and preferences of women play an important role in this decision [12, 13]. In the last decades, the number of women choosing breast reconstruction, and especially immediate breast reconstruction, has increased substantially [14–16].

Decision making regarding breast reconstruction is complex and can be challenging for women. Women often have to consider multiple options within a short and stressful period following breast cancer diagnosis, and make a decision in this timeframe that will have a lasting impact on the rest of their lives. Previous studies have highlighted the importance of the provision of high-quality, realistic preoperative information and decisional support to enable women to make a long-term satisfying decision about breast reconstruction [17–24]. Although most women are satisfied with their reconstructed breast, and decision regret is generally low [25], a minority of women experience mild to moderate levels of regret [17, 26]. Both knowledge of breast reconstruction and decisional preparedness have been shown to be low among women deciding about breast reconstruction [27–29]. A single-centre, cross-sectional study among 126 women undergoing mastectomy demonstrated that less than half of the participants made a high-quality decision regarding breast reconstruction, defined as having knowledge of important breast reconstruction facts and undergoing treatment in accordance with one’s personal preferences [30].

Patient decision aids (pDAs) are tools developed to support shared decision making between patients and physicians [31]. PDAs explicitly state the treatment decision that patients face, consist of evidence-based information about treatment options including their pros and cons, and clarify personal values relevant for the decision [31]. Across a wide variety of treatment decisions, pDAs have been found to reduce decisional conflict, increase knowledge and increase insight into personal values related to the decision, without increasing anxiety [32, 33].

Worldwide, only a limited number of interventions to support patient decision making about breast reconstruction are available [34]. In a recent review assessing the effectiveness of these interventions as compared to a control group, eight studies on seven distinct interventions were identified [34]. While the intervention improved patient satisfaction and involvement in decision making in all studies that reported on that specific outcome, results on other important outcomes were mixed [34]. In three out of five studies the intervention reduced decisional conflict [35–37], in two out of three studies the intervention reduced regret [35, 38] and in one out of three studies the intervention improved knowledge [39]. Furthermore, most included studies were rated as of weak methodological quality and none were undertaken in a European country.

To support women in making an informed decision regarding immediate breast reconstruction following mastectomy, we developed an online pDA for the Dutch population. The aim of this study is threefold. First, we aim to investigate the impact of this pDA on the decision-making process (in terms of decisional conflict, satisfaction with information, satisfaction with plastic surgeon, preparedness for decision making, perceived shared decision making, and involvement in decision making), on decision quality (in terms of knowledge of breast reconstruction and decision regret), and on patient-reported health outcomes (in terms of actual choice, satisfaction with breast, body image, sexual functioning, anxiety, and breast symptoms). Second, we aim to explore factors that are predictive of the effectiveness of the pDA. And third, the cost-effectiveness will be investigated.

## Methods/Design

### Design

We will conduct a multicenter, two-armed randomized (1:1) controlled trial. The trial protocol was retrospectively registered at the 1st of January 2019 in ClinicalTrials.gov (NCT03791138).

### Study setting

The study will be conducted in eight hospitals (two academic centres, five general hospitals and one cancer-specialized hospital) throughout the Netherlands.

### Eligibility criteria

The study sample will be composed of women (≥18 years of age or older) diagnosed with breast cancer or ductal carcinoma in situ, who will undergo mastectomy, are eligible for immediate breast reconstruction and have been referred to a plastic surgeon. The consultation with a plastic surgeon should be scheduled at least three working days after study invitation, to allow participants for sufficient time to complete informed consent (IC) and the baseline questionnaire and use the pDA or information leaflet prior to their consultation. Additionally, women must have internet access, basic computer skills and sufficient command of the Dutch language.

### Procedure

Prior to study commencement, all members of the surgical breast cancer team will receive access to the pDA and attend a meeting to familiarise with the pDA and study procedures. No further training on the delivery or use of the pDA is provided.

Women will be invited for study participation by their treating surgical oncologist, nurse specialist or breast cancer nurse during a regular pre-surgical treatment consultation in which the possibility of breast reconstruction is also discussed. The clinician will provide patients with a study information package consisting of a patient information letter and a sheet outlining patient’s treatment options that allows women to tailor the pDA to their situation (see Intervention). Patients will give written approval for sharing their contact information with the research team. A member of the research team will then contact the patient by phone to give detailed study information and to check whether inclusion criteria are met. Eligible women who are interested in participating will receive two emails, one with a link to the online IC form and one with a link to the baseline questionnaire (T0). Following completion of both, participants will be randomly allocated to the intervention or control group. Women in the intervention group will receive access to the online pDA. They will receive an email with a link to the pDA and a personal login code. Women in the control group will receive an email with a digital version of a widely available free information leaflet on breast reconstruction. Two days prior to consultation with their plastic surgeon, participants in the intervention group and the control group will be reminded by email about the possibility of using the pDA or the information leaflet respectively. Study allocation will be noted in patients’ medical records, allowing plastic surgeons to discuss the summary sheet of the pDA (see Intervention) with patients in the intervention group. Participants will complete questionnaires at T1 (1 week after consultation with the plastic surgeon), T2 (3 months after breast surgery) and T3 (12 months after breast surgery). Questionnaires will be completed online and will be available in paper format upon patient request. To minimize missing data in online questionnaires, we will mark items as obligatory. Participants will receive reminders for completing questionnaires by email up to three times. Participants allocated to the intervention group have unlimited access to the pDA during the study. Participants will not be restrained from using other information sources.

### Intervention

The online interactive pDA (named in Dutch ‘*Borstreconstructie Keuzehulp*’, translated in English as ‘Breast Reconstruction Decision Aid’) aims to support women in making a well-informed decision about breast reconstruction. It is developed to prepare women for consultation with a plastic surgeon. It contains evidence-based information about breast reconstructive options and their pros and cons. Furthermore, the pDA actively encourages women to weigh the options and discuss their preferences with their plastic surgeon during consultation.

The pDA consists of six modules: 1. Diagnosis, 2. Immediate breast reconstruction or not (yet)? 3. Expectations, 4. Considerations, 5. Patient stories, and 6. Summary (See Table 1 for a brief description of each module).

**Table 1:** Overview and brief summary of the pDA’s modules

Module	Description of module
1. Diagnosis	Based on patient’s treatment options as provided to them by their clinician during the clinical encounter, patients tailor the pDA to their situation (i.e. whether or not the patient is eligible for nipple-sparing surgery, whether or not radiotherapy is or might be necessary following surgery, and whether or not the patient is eligible for BCS).
2. Immediate reconstruction or not (yet)?	Breast reconstruction options and their pros and cons are described. Options include undergoing immediate breast reconstruction, undergoing delayed breast reconstruction, and undergoing no breast reconstruction. Information is structured as answers to the following questions: ‘What choices do I have?’, ‘What are my options?’, ‘What are the pros and cons?’, ‘How much time do I have to think?’, ‘A period without a breast?’, ‘Sparing my skin and nipple?‘^a^, ‘When can I resume my normal activities?’, ‘When is breast reconstruction finished?’, ‘What is breast-conserving surgery?‘^b^
3. Expectations	Information about what patients can expect from undergoing breast reconstruction is provided. Also, the different types of breast reconstruction and their pros and cons are described. Options include implant-based breast reconstruction and autologous breast reconstruction. Information is structured as answers to the following questions: ‘What can I expect of a new breast?’, ‘What are the pros and cons of implant-based and autologous breast reconstruction?’, ‘What if I received breast radiation in the past?’, ‘What is implant-based breast reconstruction?’, ‘What is autologous breast reconstruction?’, ‘How will my breast look like?’, ‘How will my breast feel like?’, ‘Will this impact my body image?’, ‘What are potential complications?’, ‘What if I need breast radiation following surgery?‘^c^
4. Considerations	With value clarification exercises, women are actively encouraged to weigh the options of undergoing immediate breast reconstruction or not undergoing breast reconstruction (and potentially undergoing delayed breast reconstruction). Furthermore, women are invited to indicate their breast reconstruction preference and note questions they have for their plastic surgeon.
5. Patient stories	Short stories of six women who underwent breast surgery with or without breast reconstruction. The stories illustrate the experiences of these women with decision making about breast reconstruction and the impact of their decision on their daily life.
6. Summary	A summary sheet (A4 format), including patient’s personal considerations, preferences and questions for the plastic surgeon. The sheet can be saved as PDF and printed. Patients are encouraged to discuss the summary sheet with their plastic surgeon.

^a^Text of this section is rephrased dependent on whether or not patient is eligible for nipple-sparing surgery

^b^Only shown if the patient is eligible for BCS

^c^Only shown if radiotherapy is or might be needed

The information is tailored to patient’s treatment options relevant for decision making about breast reconstruction (i.e., whether or not the patient is eligible for nipple-sparing surgery, whether or not radiotherapy is or might be necessary following surgery, and whether or not the patient is eligible for BCS). Based on these treatment options, specific information is shown or text is rephrased (See Table 1 for details). Patients can further tailor the information to their needs by selecting the information that they want to read, in their own preferred speed and order. Information is presented in a balanced way, not favouring any particular outcome. The pDA also includes illustrations of different types of breast reconstruction. It takes approximately 60 min to complete the full program. The website is secured (https) and protected by a personal login code.

#### Development of the intervention

The pDA has been developed by clinicians and researchers of the Netherlands Cancer Institute (NKI), in partnership with ZorgKeuzeLab, a company specialized in the development and implementation of decision aids. The pDA was developed in close collaboration with a multidisciplinary working group consisting of 16 professionals from seven Dutch hospitals. Furthermore, an Australian psycho-oncology researcher and health psychologist (KS), developer of the breast reconstruction decision aid (‘BRECONDA’) [36, 40, 41], contributed as a consultant.

The pDA development was guided by the criteria of the International Patient Decision Aid Standards [42], and is in line with the Dutch guideline for the development of decision aids [43]. Furthermore, it was informed by a needs assessment among women who considered breast reconstruction following mastectomy in the past and healthcare professionals. Content was created by clinicians from the NKI based on most recent guidelines [13, 44], and was critically reviewed by members of the working group. The content was rewritten to B1 language level [45] (characterized by the use of common words and short, simple and active sentences) to be understandable for most people. The technical system was created based on the existing platform of ZorgKeuzeLab.

We tested the usability of the resulting pDA among women who considered breast reconstruction following mastectomy in thepast. Furthermore, healthcare professionals and representatives of the Dutch Breast Cancer Patient Organisation, who were not involved in the development, independently reviewed the pDA. Based on received feedback, minor adaptions were made to optimize the pDA. Detailed results of the developmental process will be published.

### Control group

Patients in the control group will receive a digital version of a widely available free information leaflet about breast reconstruction developed by the Dutch Cancer Society. This information leaflet is typically included in the standard breast reconstruction information materials in Dutch hospitals. The leaflet consists of 39 pages explaining all types of breast reconstruction including drawings and photos of results. In contrast to the pDA, the leaflet is not tailored to patient’s treatment options, does not contain value clarification exercises, patient stories and a summary sheet to discuss with a plastic surgeon, and it is not structured to guide decision making.

## Study measures

### Sociodemographic and clinical data

The patient’s age, country of birth, primary language, marital status, parity, education level, work status, internet experience, hereditary breast cancer risk, history of malignancy, surgery and/or radiotherapy of the breast, neo-adjuvant treatment, surgical risk factors (i.e. weight and height, smoking status, comorbidities), eligibility for BCS and/or nipple-sparing surgery, and indication for adjuvant radiotherapy will be obtained via the baseline questionnaire. Via postsurgical follow-up questionnaires (T2 and T3), we will obtain data on surgical treatment (including type and timing of breast reconstruction, if applicable), complications and adjuvant treatment. Surgical treatment and complications will be verified against data in the electronic medical record (EMR). Date of diagnosis, tumor characteristics, second malignancies and patient’s cup size will be collected from the EMR.

### Outcome measures

An overview of outcome measures, corresponding questionnaires and measurement time points is provided in Table 2.

**Table 2:** Overview of outcome measures, corresponding instruments and measurement time points

Outcome measure	Instrument	Details	T0	T1	T2	T3
Decision-making process and decision quality						
Decisional conflict	Decisional Conflict Scale (DCS) [46, 47]	16 items, 5-point Likert-type scale, score range: 0–100, higher scores indicate more decisional conflict. Scores ≤25 are associated with follow-through decisions, and scores > 37.5 are associated with decision delay. Cronbach’s alpha = > 0.78 [47, 48]	x	x	x	x
Satisfaction with information	Three study-specific questions	How satisfied are you with the information about breast reconstruction? (5-point Likert-type scale: not at all – very satisfied), Did you miss information? (y/n), Would you have preferred less information? (y/n)			x	x
	Subscale Satisfaction with Information of the BREAST-Q [49] (slightly adapted), for women with breast reconstruction only	Satisfaction with information (Reconstruction module): 15 items, 4-point Likert-type scale, very unsatisfied – very satisfied, score range: 0–100, higher scores indicating higher satisfaction. Cronbach’s alpha = 0.94 [49]				
Satisfaction with plastic surgeon	Subscale Satisfaction with the Plastic Surgeon of the BREAST-Q [49]	Satisfaction with the Plastic Surgeon (Reconstruction module): 12 items, 4-point Likert-type scale, score range: 0–100, higher scores indicating higher satisfaction. Cronbach’s alpha = 0.97 [49]		x		
Preparedness for decision making	Preparation for Decision Making Scale [50, 51]	10 items, 5-point Likert-type scale, score range: 0–100, higher scores indicate higher perceived level of preparation for decision making. Cronbach’s alpha = 0.92–0.96 [51]		x		
Shared Decision Making	Shared Decision Making Questionnaire (SDM-Q-9) [52, 53]	9 items, 6-point Likert-type scale, score range: 0–100, higher scores indicate higher levels of perceived shared decision making. Cronbach’s alpha = 0.88 [53]		x		
Patient involvement in decision making	Control Preferences Scale [54]	1 item, 5-point Likert-type scale		x	x	
Knowledge of breast reconstruction	Study-specific questionnaire, translated and adapted from a questionnaire used in prior research [55]	10 items that can be answered with true/false/I don’t know. Items are about contraindications, risk factors, duration of the recovery period, impact of breast reconstruction on sensation, number of surgical procedures, complexity of types of breast reconstruction, complications, impact of breast reconstruction on breast cancer treatment and survival rates and the opportunity to spare the nipple. Total score is the number of correctly answered items, ranging from 0 to 10.	x	x	x	x
Decision regret	Decision Regret Scale (DRS) [56, 57]	5 items, 5-point Likert-type scale, score range: 0–100, higher scores indicating greater regret. Cronbach’s alpha = 0.81–0.92 [56]			x	x
Health outcomes						
Choice regarding breast reconstruction	Patient-reported questions and data from EMR				x	x
Satisfaction with breasts	Subscale Satisfaction with Breasts of the BREAST-Q Subscale Satisfaction with Breast Outcome of the BREAST-Q [49] (women with breast reconstruction only)	Satisfaction with Breasts (Reconstruction or Mastectomy Module, as appropriate): 16 items (women with breast reconstruction)/4 items (women without breast reconstruction)). Cronbach’s alpha = 0.96 (Reconstruction) [49]. Satisfaction with Breast Outcome (Reconstruction Module): 7 items, recall period: past 2 weeks. Higher scores indicate higher satisfaction. Cronbach’s alpha = 0.88 [49]			x	x
Body image	Subscale Body Image of the EORTC QLQ-BR23 [58]	4 items, 4-point Likert-type scale. Cronbach’s alpha = 0.69–0.91 [58]			x	x
Sexual functioning	Subscale Sexual Functioning of the EORTC QLQ-BR23 [58]	2 items + 1 item sexual enjoyment (if applicable), 4-point Likert-type scale. Cronbach’s alpha =0.87–0.94 [58]			x	X
Breast symptoms	Subscale Breast Symptoms of the EORTC QLQ-BR23 [58]	4 items, 4-point Likert-type scale. Cronbach’s alpha =0.46–0.85 [58]			x	X
Anxiety	STAI-6 (State scale of the State-Trait Anxiety Inventory) [59]	6 items, 4-point Likert-type scale,score range: 20–80, higher scores indicate higher levels of anxiety. Cronbach’s alpha = 0.82 [59]	x	x	x	x
Cost-effectiveness						
Use of health care services	A selection of questions of the Medical Consumption Questionnaire (see https://www.imta.nl/), and data from EMR	Selection of questions on the number of consultations related to breast surgery with a (plastic) surgeon, nurse practitioner/ nurse specialist, social worker, psychologist, general practitioner and a physiotherapist, and the amount of received home care during the last 3 months (T2) or 9 months (T3).			x	x
Health-Related Quality of life	EuroQoL-5D-5 L [60]	EuroQoL-5D-5 L descriptive system: 5 items, 5-point Likert-type scale, and the EQ Visual Analogue Scale: patients’ self-rated health.	x		x	x

#### Main outcome

The primary outcome is decisional conflict, measured by the Decisional Conflict Scale (DCS) [46]. Decisional conflict is defined as a state of uncertainty about the course of action to take [61]. The DCS measures how well-informed patients feel about their decision, the level of uncertainty about the best choice, and the perceived effectiveness of decision making. It has a total scale and five subscales (uncertainty, feeling informed, feeling clear about values, feeling supported and effective decision-making). Items belonging to the subscale effective decision-making will not be assessed at baseline, since these items were considered inappropriate to assess before patients had a consultation with a plastic surgeon. The DCS is reliable and valid [46–48], and is the most commonly used instrument to evaluate effectiveness of pDAs [62].

#### Secondary outcomes

##### Decision-making process and decision quality

The decision-making process is further measured in terms of I) satisfaction with information [49], II) satisfaction with the plastic surgeon [49], III) preparedness for decision making [50, 51], IV) patients’ perceived levels of shared decision making during consultation with their plastic surgeon [52, 53], and V) patients’ perceived level of involvement in decision making [54]. Decision quality is measured by I) knowledge of breast reconstruction [36, 55] and II) decision regret [56, 57].

##### Patient-reported health outcomes

Patients’ actual choice regarding breast reconstruction will be measured by patient-report at postsurgical follow-up (T2, T3), and will be verified against patients’ EMR. Patient satisfaction with the breast [49], body image [58], sexual functioning [58], and breast symptoms [58] will be obtained at postsurgical follow-up. Anxiety will be measured at all four time points [59].

### Moderating measures

At baseline, we will measure patients’ preferred level of involvement in decision making regarding breast reconstruction (Control Preferences Scale [54]), preference for breast reconstruction (1 item, 5 point Likert-type Scale, with 1 = “I have a strong preference for undergoing breast reconstruction”, and 5 =“I have a strong preference for not undergoing breast reconstruction”) and information coping style (Threatening Medical Situations Inventory [63]). At all assessments, patients will be asked to report on the information sources they used regarding breast reconstruction.

### Process measures

Among women in the intervention group, the actual usage of the pDA (i.e. whether and when they logged in, whether the summary sheet was discussed with a plastic surgeon) will be obtained via self-report at T1. Usage data will be supplemented with log data collected in the pDA (e.g., number of logins, number of completed modules). Additionally, at T1, all participants will report on how satisfied they are with the received information (i.e., pDA or information leaflet) and how useful it was for them in making a decision about breast reconstruction.

### Cost-effectiveness

For the cost-effectiveness analysis we will determine incremental costs, incremental effects (in terms of quality adjusted life years (QALYs), reduction in decisional conflict, reduction of regret), and the incremental cost-effectiveness ratio.

#### Utilities

QALYs are calculated by multiplying the life years with the utilities. An utility is a score that is derived from the generic five-level EuroQol five-dimensional questionnaire (EQ-5D-5 L) [60]. This preference based instrument consists of five dimensions: mobility, self-care, usual activities, pain/discomfort and anxiety/depression [60, 64].

#### Costs

Unit costs will be estimated based on the trial data and published sources in the Netherlands [65]. Fixed costs of the development of the pDA will be based on the R&D of the pDA, including expected maintenance costs. For the control group, costs of a leaflet will be taken into account. Direct medical costs will include (1) treatment costs: type and number of (reconstructive) breast surgeries during study participation (data collected from the EMR), number of nights hospitalized (EMR)/inpatient days in hospital (for any reason) (EMR), and (2) resource use: participants are asked for professional care resources they used related to their breast (reconstructive) surgery. This consists of the number of consultations (live or by phone) with plastic surgeons and other medical and paramedical professionals pre- and post-operatively. For medical consumption, patients are asked to report on the received amount of home care (selection of items of the iMTA Medical Consumption Questionnaire on T2 3 months after surgery and T3 12 months after surgery; see https://www.imta.nl/). Production losses are measured by means of work status at T2 and T3, controlled for work status at baseline, and the number of hours in sick leave.

### Randomization

Simple randomization stratified by site and patients’ surgical treatment options (e.g., whether the patient is eligible for both mastectomy and BCS or for mastectomy only) will be used to assign participants to the intervention group or the control group. Randomization ratio is 1:1. Randomization will be performed with ALEA software. This study is non-blinded, since blinding was not considered feasible due to the nature of the trial.

### Statistical analyses

Data will be pseudonymized prior to data analysis and will not be traceable to any individuals. Depending on the level of measurement, analysis of variance or appropriate non-parametric statistics will be used to evaluate the comparability of baseline sociodemographic and clinical characteristics between the intervention and the control group. If differences in background characteristics are found, these will be taken into account in the subsequent analyses. Scores on the questionnaires will be calculated according to published scoring algorithms.

We will look at group differences in decisional conflict for the entire study duration (T0 to T3) using a mixed effect growth model with random intercept and slope and site as a cluster variable. This approach takes into account the within and between person variability, and deals adequately with missing data [66]. A comparable mixed effect model approach will also be used to determine the effects of the pDA on other patient reported outcomes. Differences in mean change scores over time between the intervention and control group will be accompanied by effect sizes (ES). An ES of 0.2 is considered small, 0.5 moderate and 0.8 large [67].

Analyses will be done on an intention-to-treat basis. As a secondary analysis, per-protocol analyses will be carried out on data from patients who meet the criteria for minimal compliance (to be determined). Appropriate statistical measures will be taken to adjust for multiple comparisons.

To evaluate between-group differences over time in categorical variables such as the actual choice in breast reconstruction, generalized estimating equations for longitudinal data will be used. This approach accounts for correlated within subject responses, allows for not normally distributed variables and deals adequately with missing data [68, 69].

We will explore which variables are predictive for the efficacy of the pDA on the primary outcome (decisional conflict) within the intervention group. A mixed effect model for longitudinal data will be used with decisional conflict as dependent variable and the following independent (baseline) variables: knowledge of breast reconstruction, patients’ preferred level of involvement in decision making, information coping style, and sociodemographic variables. The *p*-values for each model will be adjusted for multiple comparisons.

### Cost-effectiveness analysis

We will perform a cost-effectiveness analysis comparing the pDA with usual care expressed as: (1) cost per clinically relevant reduction in decision regret (as measured by the Decision Regret Scale (DRS) [57]), (2) cost per clinically relevant reduction in decisional conflict (as measured by the DCS [47]), and (3) cost per QALY gained.

For decision regret, measured with the DRS, we will use a score of 30 out of 100 as a cut-off point [70]. A score of 30 or higher means that a participant responded that she was more or less in agreement with at least one of the statements about an experience of regret [70]. For decisional conflict, measured with the DCS, we will use a score of 37.5 out of 100 as a cut-off point [47, 71–73]. Scores exceeding 37.5 are associated with decision delay and feeling unsure about implementation [46, 47].

A Markov model will be constructed with four mutually exclusive health states: “no regret”, “regret”,“recurrence” and “death” (or “no decisional conflict”, “decisional conflict”, “recurrence” and “death”). A healthcare and societal perspective from the Netherlands, plus a 5 year time horizon [25], and a cycle length of 3 months will be adopted. Production losses will be analysed by means of the Friction cost method [74]. The friction cost method calculates the costs over the friction period; the period in which the patient has not yet been replaced at work by another employee. Future costs and effects will be discounted at 4 and 1.5%, respectively, in line with Dutch guidelines [65]. The incremental costs-effectiveness ratio is calculated by dividing the difference in total costs of pDA and usual care by the difference in (1) reduction of regret/decisional conflict and (2) QALYs, which indicates the additional costs of pDA per QALY gained. The deterministic mean together with the degree of uncertainty, represented by the relevant distributions around the input parameters, will be estimated. Sensitivity analyses will be carried out to test the robustness of the model. Probabilistic sensitivity analyses will be performed to estimate the probability of cost-effectiveness. Visualization of data will be realized by means of a cost-effectiveness plane and cost-effectiveness acceptability curve [75, 76]. A ceiling ratio of €20.000/QALY, corresponding with the Dutch threshold for willingness to pay, will be used in this analysis [77].

### Power calculation

Power calculations for estimating sample size requirements were based on the following criteria: (1) power of 0.80, (2) alpha of 0.05, and (3) an ES d of 0.4. With these criteria a total sample size of 198 cases (99 per group) is needed. To allow for an anticipated attrition rate of approximately 20%, we will recruit 250 participants.

## Discussion

Decision making about breast reconstruction is challenging and the preference-sensitive nature of this decision requires an approach of shared decision making between patient and physician. To support women with breast cancer in making a well-informed decision about immediate breast reconstruction and optimize the decision-making process, we developed an online pDA. We hypothesize that the pDA will improve the decision-making process, the decision quality and health outcomes. This study will provide valuable insights into the impact of an online decision support tool for an increasing group of women facing the choice for immediate breast reconstruction after mastectomy.

Our study has several strengths. First, in evidence-based research, randomized controlled trials are considered to produce the highest level of evidence when evaluating the effectiveness of interventions [78, 79] . Second, assessments are at clinical relevant time points and include longer follow-up than in previous studies [34, 37–39]. Since the process of breast reconstruction can take up to 1 year or longer and outcomes only become evident after a while [25, 80], our study will give a more accurate account of this process and the different issues surrounding it. Finally, a cost-effectiveness analysis will provide new insights into the added value of the pDA in terms of cost-effectiveness [32, 81].

There are also some limitations to our current study. First, its design may lead to an underestimation of the pDA’s impact. By providing the information leaflet to women in the control group, the control group can partially be considered as an active control group, and the effects of the pDA on outcomes such as knowledge might be reduced. However, we provided the information leaflet to the control group for ethical reasons and we expect that it will stimulate recruitment and decrease drop-out rates in the control group, as was suggested in a study in which 27% of enrolled participants dropped out because they refused to participate when they were randomized to the control group without any additional information [39]. Secondly, there is a potential risk of contamination caused by the individual randomisation. Although there is little empirical evidence that contamination is a real problem for trials on educational interventions [82], it seems plausible that plastic surgeons adjust the style, structure and/or content of their consultations based on their experiences with women in the intervention group, after reviewing the pDA itself, or simply by participating in the trial. Cluster randomization to minimize contamination was however considered less favourable due to problems with selection bias and the need for larger samples [83].

Because of an increasing number of women who are offered immediate breast reconstruction and the clearly expressed need for decision support by women facing this complex decision, our pDA is expected to fill a gap in current clinical practice. This study contributes to the knowledge of the impact of a pDA on the decision-making process and decision quality. If the pDA positively contributes to the decision-making process and the decision quality, the pDA can be implemented nationwide.

## Data Availability

The dataset generated in the current study will be available from the corresponding author (stored in a data repository at the Netherlands Cancer Institute) on reasonable request.

## Abbreviations

BCS: Breast-Conserving Surgery
DCS: Decisional Conflict Scale
DRS: Decision Regret Scale
EMR: Electronic Medical Record
ES: Effect Size
IC: Informed Consent
IPDAS: International Patient Decision Aid Standards
NKI: Netherlands Cancer Institute
pDA: Patient Decision Aid
QALYs: Quality Adjusted Life Years

## References

[1] Dutch Cancer Registry. https://www.cijfersoverkanker.nl/. Accessed 18 Mar 2019.

[2] Global Cancer Observatory. http://gco.iarc.fr/. Accessed 20 Oct 2018.

[3] Curado MP, Edwards B, Shin HR, Storm H, Ferlay J, Heanue M (2007). Cancer incidence in five continents, volume IX.

[4] Jeevan R, Cromwell DA, Browne JP, Caddy CM, Pereira J, Sheppard C (2014). Findings of a national comparative audit of mastectomy and breast reconstruction surgery in England. J Plast Reconstr Aesthet Surg.

[5] Roder D, Zorbas H, Kollias J, Pyke C, Walters D, Campbell I (2013). Factors predictive of immediate breast reconstruction following mastectomy for invasive breast cancer in Australia. Breast.

[6] National Breast and Ovarian Cancer Centre (2010). National Breast and Ovarian Cancer Centre and Royal Australasian College of Surgeons National Breast Cancer Audit Public Health Monitoring Series 2008 Data.

[7] NABON Breast Cancer Audit. NBCA Annual Report 2017. Available from: https://dica.nl/jaarrapportage-2017. Accessed 7 Jan 2019.

[8] Parker PA, Youssef A, Walker S, Basen-Engquist K, Cohen L, Gritz ER (2007). Short-term and long-term psychosocial adjustment and quality of life in women undergoing different surgical procedures for breast cancer. Ann Surg Oncol.

[9] Koçan S, Gürsoy A (2016). Body image of women with breast cancer after mastectomy: a qualitative research. J Breast Health.

[10] Janni W, Rjosk D, Dimpfl T, Haertl K, Strobl B, Hepp F (2001). Quality of life influenced by primary surgical treatment for stage I-III breast cancer-long-term follow-up of a matched-pair analysis. Ann Surg Oncol.

[11] Chen CL, Liao MN, Chen SC, Chan PL, Chen SC (2012). Body image and its predictors in breast cancer patients receiving surgery. Cancer Nurs.

[12] Lee GK, Sheckter CC (2018). Breast reconstruction following breast cancer treatment-2018. JAMA..

[13] Dutch Society for Plastic Surgery. Guideline Breast Reconstruction (2015). www.nvpc.nl. Accessed Oct 2016.

[14] Panchal H, Matros E (2017). Current trends in postmastectomy breast reconstruction. Plast Reconstr Surg.

[15] Mennie JC, Mohanna PN, O’Donoghue JM, Rainsbury R, Cromwell DA (2017). National trends in immediate and delayed post-mastectomy reconstruction procedures in England: a seven-year population-based cohort study. Eur J Surg Oncol.

[16] van Bommel Annnelotte, Spronk Pauline, Mureau Marc, Siesling Sabine, Smorenburg Carolien, Tollenaar Rob, Vrancken Peeters Marie-Jeanne, van Dalen Thijs (2019). Breast-Contour-Preserving Procedure as a Multidisciplinary Parameter of Esthetic Outcome in Breast Cancer Treatment in The Netherlands. Annals of Surgical Oncology.

[17] Sheehan J, Sherman KA, Lam T, Boyages J (2007). Association of information satisfaction, psychological distress and monitoring coping style with post-decision regret following breast reconstruction. Psychooncology..

[18] Dikmans REG, van de Grift TC, Bouman MB, Pusic AL, Mullender MG (2019). Sexuality, a topic that surgeons should discuss with women before risk-reducing mastectomy and breast reconstruction. Breast.

[19] Kuo NT, Kuo YL, Lai HW, Ko NY, Fang SY (2019). The influence of partner involvement in the decision-making process on body image and decision regret among women receiving breast reconstruction. Support Care Cancer.

[20] Lee CN, Pignone MP, Deal AM, Blizard L, Hunt C, Huh R (2018). Accuracy of predictions of patients with breast cancer of future well-being after immediate breast reconstruction. JAMA Surg..

[21] Hasak JM, Myckatyn TM, Grabinski VF, Philpott SE, Parikh RP, Politi MC (2017). Stakeholders’ perspectives on postmastectomy breast reconstruction: recognizing ways to improve shared decision making. Plast Reconstr Surg Glob Open.

[22] Zhong T, Hu J, Bagher S, O'Neill AC, Beber B, Hofer SO (2013). Decision regret following breast reconstruction: the role of self-efficacy and satisfaction with information in the preoperative period. Plast Reconstr Surg.

[23] Potter S, Mills N, Cawthorn S, Wilson S, Blazeby J (2015). Exploring information provision in reconstructive breast surgery: a qualitative study. Breast.

[24] Soon PS, Ruban S, Mo HTJ, Lee R, Saliba L, Shah A (2019). Understanding patient choices regarding breast reconstruction after mastectomy for breast cancer. Support Care Cancer.

[25] Flitcroft K, Brennan M, Spillane A (2018). Decisional regret and choice of breast reconstruction following mastectomy for breast cancer: a systematic review. Psychooncology..

[26] Zhong T, Bagher S, Jindal K, Zeng D, O'Neill AC, MacAdam S (2013). The influence of dispositional optimism on decision regret to undergo major breast reconstructive surgery. J Surg Oncol.

[27] Manne SL, Topham N, Kirstein L, Virtue SM, Brill K, Devine KA (2016). Attitudes and decisional conflict regarding breast reconstruction among breast cancer patients. Cancer Nurs.

[28] Fallbjörk U, Frejeus E, Rasmussen BH (2012). A preliminary study into women's experiences of undergoing reconstructive surgery after breast cancer. Eur J Oncol Nurs.

[29] Murray CD, Turner A, Rehan C, Kovacs T (2015). Satisfaction following immediate breast reconstruction: experiences in the early post-operative stage. Br J Health Psychol.

[30] Lee CN, Deal AM, Huh R, Ubel PA, Liu YJ, Blizard L (2017). Quality of patient decisions about breast reconstruction after mastectomy. JAMA Surg.

[31] Joseph-Williams N, Newcombe R, Politi M, Durand MA, Sivell S, Stacey D (2014). Toward minimum standards for certifying patient decision aids: a modified delphi consensus process. Med Decis Mak.

[32] Stacey D, Légaré F, Lewis K, Barry MJ, Bennett CL, Eden KB (2017). Decision aids for people facing health treatment or screening decisions. Cochrane Database Systemat Rev.

[33] Sheehan J, Sherman KA (2012). Computerised decision aids: a systematic review of their effectiveness in facilitating high-quality decision-making in various health-related contexts. Patient Educ Couns.

[34] Paraskeva N, Guest E, Lewis-Smith H, Harcourt D (2018). Assessing the effectiveness of interventions to support patient decision making about breast reconstruction: a systematic review. Breast.

[35] Lam WW, Chan M, Or A, Kwong A, Suen D, Fielding R (2013). Reducing treatment decision conflict difficulties in breast cancer surgery: a randomized controlled trial. J Clin Oncol.

[36] Sherman KA, Shaw LK, Winch CJ, Harcourt D, Boyages J, Cameron LD (2016). Reducing decisional conflict and enhancing satisfaction with information among women considering breast reconstruction following mastectomy: results from the BRECONDA randomized controlled trial. Plast Reconstr Surg.

[37] Causarano N, Platt J, Baxter NN, Bagher S, Jones JM, Metcalfe KA (2015). Pre-consultation educational group intervention to improve shared decision-making for postmastectomy breast reconstruction: a pilot randomized controlled trial. Support Care Cancer.

[38] Luan A, Hui KJ, Remington AC, Liu X, Lee GK (2016). Effects of a novel decision aid for breast reconstruction: a randomized prospective trial. Ann Plast Surg.

[39] Heller L, Parker PA, Youssef A, Miller MJ (2008). Interactive digital education aid in breast reconstruction. Plast Reconstr Surg.

[40] Sherman KA, Harcourt DM, Lam TC, Shaw LK, Boyages J (2014). BRECONDA: development and acceptability of an interactive decisional support tool for women considering breast reconstruction. Psychooncology..

[41] Sherman KA, Shaw LK, Jørgensen L, Harcourt D, Cameron L, Boyages J (2017). Qualitatively understanding patients’ and health professionals’ experiences of the BRECONDA breast reconstruction decision aid. Psychooncology..

[42] Elwyn G, O’Connor AM, Bennett C, Newcombe RG, Politi M, Durand MA (2009). Assessing the quality of decision support technologies using the international patient decision aid standards instrument (IPDASi). PLoS One.

[43] Netherlands Patients Federation, Dutch College of General Practitioners, Royal Dutch Medical Association, Dutch Professional Nurses Organisation (2018). Guideline decision aid with guidelines [in Dutch].

[44] National Breast Cancer Network Netherlands (NABON) (2012). Breast Cancer Guideline. www.oncoline.nl. Accessed Oct 2016.

[45] Council of Europe (2001). Common European framework of reference for languages: learning, teaching, assessment.

[46] O'Connor AM (1995). Validation of a decisional conflict scale. Med Decis Mak.

[47] O’Connor AM (1993). User manual - decisional conflict scale.

[48] Koedoot N, Molenaar S, Oosterveld P, Bakker P, de Graeff A, Nooy M (2001). The decisional conflict scale: further validation in two samples of Dutch oncology patients. Patient Educ Couns.

[49] Pusic AL, Klassen AF, Scott AM, Klok JA, Cordeiro PG, Cano SJ (2009). Development of a new patient-reported outcome measure for breast surgery: the BREAST-Q. Plast Reconstr Surg.

[50] Graham ID, O’Connor AM (1995). User manual – preparation for decision making scale.

[51] Bennett C, Graham ID, Kristjansson E, Kearing SA, Clay KF, O'Connor AM (2010). Validation of a preparation for decision making scale. Patient Educ Couns.

[52] Kriston L, Scholl I, Holzel L, Simon D, Loh A, Harter M (2010). The 9-item shared decision making questionnaire (SDM-Q-9). Development and psychometric properties in a primary care sample. Patient Educ Couns.

[53] Rodenburg-Vandenbussche S, Pieterse AH, Kroonenberg PM, Scholl I, van der Weijden T, Luyten GP (2015). Dutch translation and psychometric testing of the 9-item shared decision making questionnaire (SDM-Q-9) and shared decision making questionnaire-physician version (SDM-Q-doc) in primary and secondary care. PLoS One.

[54] Degner LF, Sloan JA, Venkatesh P (1997). The control preferences scale. Can J Nurs Res.

[55] Sherman KA, Kilby CJ, Shaw LK, Winch C, Kirk J, Tucker K (2017). Facilitating decision-making in women undergoing genetic testing for hereditary breast cancer: BRECONDA randomized controlled trial results. Breast.

[56] Brehaut JC, O'Connor AM, Wood TJ, Hack TF, Siminoff L, Gordon E (2003). Validation of a decision regret scale. Med Decis Mak.

[57] O’Connor AM (1996). User manual – decision regret scale.

[58] Sprangers MA, Groenvold M, Arraras JI, Franklin J, te Velde A, Muller M (1996). The European organization for research and treatment of cancer breast cancer-specific quality-of-life questionnaire module: first results from a three-country field study. J Clin Oncol.

[59] Marteau TM, Bekker H (1992). The development of a six-item short-form of the state scale of the Spielberger state-trait anxiety inventory (STAI). Br J Clin Psychol.

[60] EuroQol Group (1990). EuroQol--a new facility for the measurement of health-related quality of life. Health Policy.

[61] O’Connor AM, GK MF, EA MF (1993). Decisional conflict. Nursing: Diagnosis and Intervention.

[62] Sepucha KR, Borkhoff CM, Lally J, Levin CA, Matlock DD, Ng CJ (2013). Establishing the effectiveness of patient decision aids: key constructs and measurement instruments. BMC Med Inform Decis Mak.

[63] van Zuuren FJ, de Groot KI, Mulder NL, Muris P (1996). Coping with medical threat: an evaluation of the threatening medical situations inventory (TMSI). Pers Individ Dif.

[64] Dolan P (1997). Modeling valuations for EuroQol health states. Med Care.

[65] Hakkaart-van Roijen L, Van der Linden N, Bouwmans CAM, Kanters T, Tan SS (2015). Costing manual: methodology of costing research and reference prices for economic evaluations in healthcare [in Dutch].

[66] Diggle PJ, Heagerty P, Liang KY, Zeger, SL. Analysis of Longitudinal Data. 2nd ed. New York: Oxford University Press; 2002, pp 169–189.

[67] Cohen J (1998). Statistical power analysis for the behavioral sciences.

[68] Ghisletta Paolo, Spini Dario (2004). An Introduction to Generalized Estimating Equations and an Application to Assess Selectivity Effects in a Longitudinal Study on Very Old Individuals. Journal of Educational and Behavioral Statistics.

[69] Liu Ivy, Agresti Alan (2005). The analysis of ordered categorical data: An overview and a survey of recent developments. Test.

[70] Becerra Perez MM, Menear M, Brehaut JC, Légaré F (2016). Extent and predictors of decision regret about health care decisions: a systematic review. Med Decis Mak.

[71] Lalonde L, O'Connor AM, Duguay P, Brassard J, Drake E, Grover SA (2006). Evaluation of a decision aid and a personal risk profile in community pharmacy for patients considering options to improve cardiovascular health: the OPTIONS pilot study. Int J Pharm Pract.

[72] Patel SR, Wisner KL (2011). Decision making for depression treatment during pregnancy and the postpartum period. Depress Anxiety.

[73] Labrecque M, Paunescu C, Plesu I, Stacey D, Légaré F (2010). Evaluation of the effect of a patient decision aid about vasectomy on the decision-making process: a randomized trial. Contraception..

[74] Garrison LP, Mansley EC, Abbott TA, Bresnahan BW, Hay JW, Smeeding J (2010). Good research practices for measuring drug costs in cost-effectiveness analyses: a societal perspective: the ISPOR drug cost task force report--part II. Value Health.

[75] Fenwick E, Claxton K, Sculpher M (2001). Representing uncertainty: the role of cost-effectiveness acceptability curves. Health Econ.

[76] Briggs AH, Claxton K, Sculpher MJ (2006). Decision modelling for health economic evaluation.

[77] Council for Public Health and Care (RVZ) (2006). Sensible and sustainable care [in Dutch]. Available from: www.raadrvs.nl/documenten/publicaties/2006/06/07/zinnige-en-duurzame-zorg. Accessed 24 Jan 2019.

[78] Centre for Evidence-Based Medicine. www.cebm.net. Accessed 10 May 2019.

[79] Campbell M, Fitzpatrick R, Haines A, Kinmonth AL, Sandercock P, Spiegelhalter D (2000). Framework for design and evaluation of complex interventions to improve health. BMJ..

[80] Breast Reconstruction Surgery. www.cancer.org/cancer/breast-cancer/reconstruction-surgery.html. Accessed 2 Apr 2019.

[81] Trenaman L, Bryan S, Bansback N (2014). The cost-effectiveness of patient decision aids: a systematic review. Healthcare..

[82] Keogh-Brown MR, Bachmann MO, Shepstone L, Hewitt C, Howe A, Ramsay CR, et al. Contamination in trials of educational interventions. Health Technol Assess. 2007;11(43):1–130. 10.3310/hta11430 17935683

[83] Puffer S, Torgerson D, Watson J (2003). Evidence for risk of bias in cluster randomised trials: review of recent trials published in three general medical journals. BMJ..

